# Rate-dependent interface capture beyond the coffee-ring effect

**DOI:** 10.1038/srep24628

**Published:** 2016-04-19

**Authors:** Yanan Li, Qiang Yang, Mingzhu Li, Yanlin Song

**Affiliations:** 1 Key Laboratory of Green Printing, Institute of Chemistry, Chinese Academy of Sciences (ICCAS), Beijing Engineering Research Center of Nanomaterials for Green Printing Technology, Beijing National Laboratory for Molecular Sciences (BNLMS), Beijing, 100190, P. R. China; 2 University of Chinese Academy of Sciences, Beijing, 100049, P. R. China

## Abstract

The mechanism of droplet drying is a widely concerned fundamental issue since controlling the deposition morphology of droplet has significant influence on printing, biology pattern, self-assembling and other solution-based devices fabrication. Here we reveal a striking different kinetics-controlled deposition regime beyond the ubiquitous coffee-ring effect that suspended particles tend to kinetically accumulate at the air-liquid interface and deposit uniformly. As the interface shrinkage rate exceeds the particle average diffusion rate, particles in vertical evaporation flow will be captured by the descending surface, producing surface particle jam and forming viscous quasi-solid layer, which dramatically prevents the trapped particles from being transported to drop edge and results in uniform deposition. This simple, robust drying regime will provide a versatile strategy to control the droplet deposition morphology, and a novel direction of interface assembling for fabricating superlattices and high quality photonic crystal patterns.

The evaporation of solvent brings about not only the concentration of solute, but also the spatial redistribution of the dispersed phase^1^,^2^,^3^,^4^,^5^,^6^. Drying of droplet is actually a complex, non-equilibrium and difficult-to-control process^4^, despite the controlling the deposition morphology of droplet has significant influence on printing^7^,^8^, biology pattern^9^,^10^,^11^ self-assembling^8^,^12^ and other solution-based devices fabrication^13^,^14^,^15^,^16^. When a drop of coffee dries on a substrate, it often leaves a ring-like stain, known as the coffee-ring effect^1^. This ubiquitous phenomenon appears when the drop contact line remains pinned during the drying process; the suspended particles tend to accumulate at the drop edge for capillary outflow to replenish the local rapid solvent loss. Further studies on this nonuniform redistribution process indicate that inner flows, including capillary flow^2^ and Marangoni flow^3^,^11^,^17^, dynamics of the three-phase contact line^6^,^7^,^8^,^13^,^18^,^19^, and particle-particle/particle-interface interaction^4^,^20^,^21^,^22^ will influence the final particle distribution. However, to study the initial mechanism of a drying droplet, much of the physics of the coffee-ring effect is demonstrated by drying the aqueous droplet in an open space at room temperature. In this condition, the distinct evaporation difference between drop edge and drop center is hard to avoid, droplet usually dries at relatively slow evaporation rate that most of the suspended particles are transported to drop edge by the strong capillary outflow (Fig. 1a).

## Results

**Figure 1:**
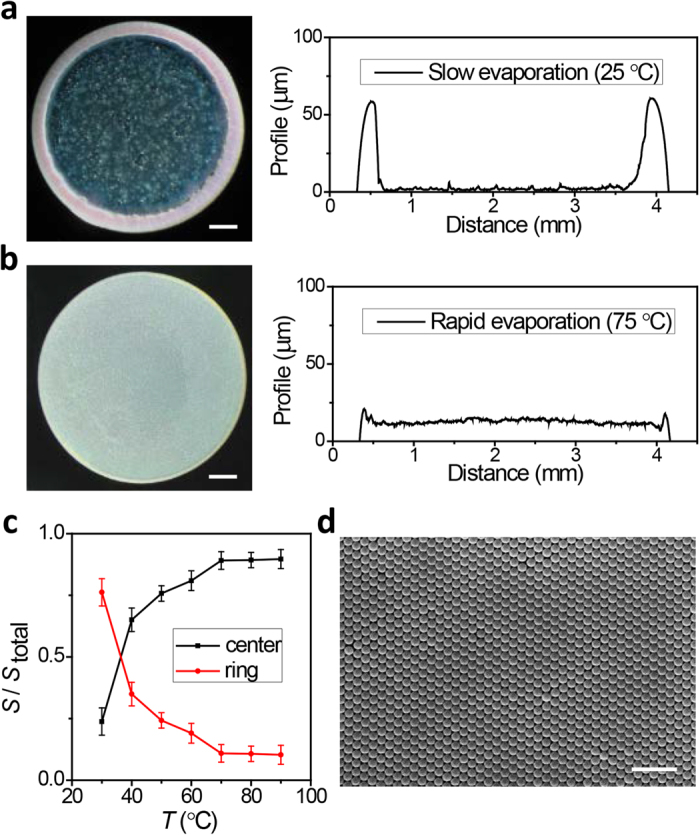
The suppressed coffee ring effect with increasing evaporation rate. **(a**,**b**) Images and morphology profiles of the final deposition by drying at room temperature (**a**, *T* = 25 °C) and high temperature (**b**, *T* = 75 °C) with constant humidity (50%). Most of the particles deposit at drop edge forming ring-like fashion when drying at slow evaporation rate. In contrast, a uniform deposition is formed with only a tiny part of the particle deposited on edge when drying at high temperature. (**c**) The cross section area proportion (*S/S*_*total*_) of ring region decreases as that of the center region arises when the evaporation temperature rises, indicating the gradually suppression of coffee-ring effect. (**d**) Highly ordered assembling of the monodispersed particles. The scale bar, 0.5 mm in (**a**,**b**), 1 μm in (**d)**.

In this work, we find that if the droplet dries rapid enough, particles tends to accumulate at the air-liquid interface rather than at the drop edge and directly deposit in the interior (Fig. 1b, Mov. S1, S2). Thus, uniform deposition can be achieved by such a simple and universal evaporation kinetics control without changing the droplet composition^3^,^5^ or particles modification^4^,^23^.

We carry out our experiment by drying the droplet in a chamber with precise temperature and humidity control (constant temperature humidity chamber, CTHC)—ranging from room temperature to near boiling point of water (90 °C) with constant humidity (50%)—to study the drying behavior at different evaporation rate. Water drops containing monodispersed hydrophilic polystyrene nanospheres^24^ (diameter 216 nm) evaporate on untreated clean silicon wafer with contact angle about 43°. We study mass fraction (*ω*) varying from 10^−1^ to 10^−4^ and droplet sizes (*V*) vary from 100 μL to 0.5 μL. Similar results are obtained for other particles with hydrophobic surface or polydispersed size (Fig. S1).

Profile of each deposition obtained from different drying conditions demonstrates the evolution of the gradually suppressed coffee-ring effect, growth of the center-region and consequent diminution of the ring-region. In order to quantify the behavior shown in Fig. 1a,b, we determine the cross section area proportion of the ring-region and interior-region in final deposition (Fig. 1c). Drying at low temperature (30 °C), near 90% of the suspended particles accumulate at drop edge and form ring-like fashion. As the temperature arises, more particles deposit in drop center and the areal fraction of the ring-region decreases in contrary. These results indicate that the deposition morphology could be changed by evaporation temperature. More interestingly, SEM image shows the particle assembles well orderly, which shows great potential for patterned photonic crystal films^25^,^26^,^27^,^28^ (Fig. 1d).

**Figure 2:**
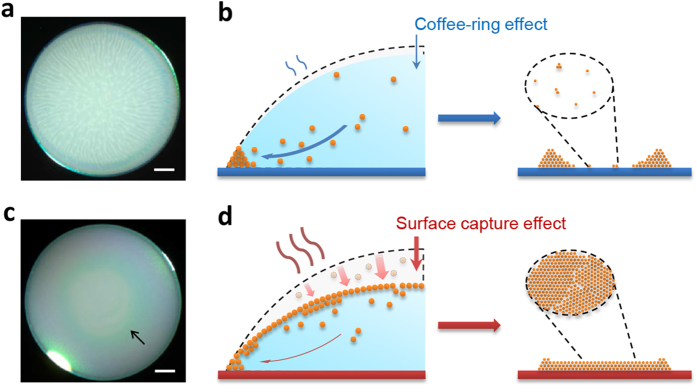
Two regimes of drying process controlled by evaporation kinetics. **(a**,**b**) Obvious capillary outflow (streamlines in snapshot a) transport the suspended particles to drop edge at low evaporation temperature (*T* = 30 °C), forming ring stains (schematic **b**). (**c**,**d**) Iridescence on drop surface at high temperature (*T* = 70 °C, arrowed in **c**) indicates the assembling of on drop surface by an collection process, forming uniform deposition (schematic **d**). Particles near surface (shaded particles in the gray area in **d** left) are captured by the rapid descending surface with only a tiny part been transported to drop edge. The thickness of the outflow arrows schematic the relative intensity. The scale bar, 0.5 mm in (**a**,**c)**.

Why particles tend to deposit at center when the evaporation temperature increases? We initially deduced that the suppressed coffee-ring effect might be caused by the thermal Marangoni flow because of enlarged temperature difference between drop edge and drop apex on hot plate (Fig. S2) However, previous literatures have reported that the Marangoni flow is weak in water droplet^3^,^29^, and our experiments show that deposition is more uniform in CTHC than that on the hot plate (Figs S3 and S4, Mov. S3, S4), which indicates the temperature caused Marangoni flow has weak influence on the deposition morphology. To further reveal the mechanism, we use stereomicroscope to investigate different drying behaviors of droplets with pinned contact line (Mov. S1, S2). When a drop dries at near room temperature (30 °C), obvious outflow induces edge growth process and forms ring-like fashion (Fig. 2a,b). In contrast, a drop drying on heated substrate (70 °C) shows quite inconspicuous growth of the ring region. Interestingly, some angle-dependent iridescence emerges on the surface, which is absence at low temperature (Fig. 2c). This iridescence lasts long until the end of the drying process, and it appears earlier when increasing the substrate temperature. As no pigment was added, we will find that this luster comes from the structure color of ordered assembling of the suspended monodispersed polystyrene particles on drop surface, like color and luster of opal^30^. This surface assembling process produces increased surface viscosity that is much larger than the bulk^4^, preventing the suspended particles at air-water interface from moving to the contact line (Fig. 2d).

As the influence of thermal Marangoni flow shows to be tiny, we speculate that this suppressed coffee-ring phenomenon may stem from a surface accumulation process caused by rate-controlled interface capture effect^31^,^32^,^33^,^34^, similar to surface skinning of drying paint and milk. Namely, particles can accumulate at the air-liquid interface by kinetically evaporation. When the interface descends more quickly than particle diffusion, particles will be captured by the interface^12^, forming a quasi-steady-state semi-solid layer, which prevents the particles from transporting to the drop edge. Just like fishing, if the net closes so fast that there is little time for the fishes to escape, they will be captured by the net.

**Figure 3:**
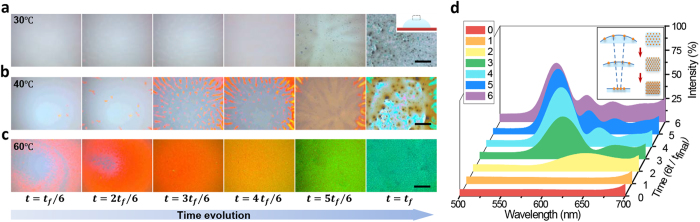
Surface assembling evolution by rate-dependent interface interception. **(a)** Absent surface accumulation at low evaporation temperature. (**b**) A few isolated particle islands appear on surface when drying at relative high temperature (40 °C). As the evaporation rate is still slow, most islands disappeared at the late evaporation stage, leaving part of bare substrate. (**c**) With further temperature increasing (60 °C), particle islands arise soon and quickly integrate together, forming a continuous particle layer on the whole drop surface. (**d**) Real-time reflection spectra of the surface assembling in (**c)**. The blue shift of surface color origins from the shrinkage of particle interspace when the drop surface varies from sphere cap to flat (inset in **d**). All the snapshots are captured on region about 1 mm at drop apex (inset in **a**). The scale bar, 150 μm in (**a**–**c)**.

Snapshots of region at drop apex (Fig. 3a inset) confirm this surface capture phenomenon. No visible particles assemble on drop surface when drying at low temperature because of slow evaporation rate (Fig. 3a, 30 °C), instead most particles are transported to contact line (Mov. S1). As the evaporation temperature rising, the air-liquid interface descends sharply and a few particle islands come into view (Fig. 3b). However, the interface-descending rate is still too slow to form larger domains, much less incorporate the isolate islands together to form a continuous particle layer. In addition, due to the insufficient particle supply and spontaneous desorption from high concentration near surface to bulk, the formed islands disappear at later stage of evaporation because of the weak interaction between isotropic spherical particles and air-liquid interface^4^,^35^ fail to fix the captured particles firmly. Therefore, coffee-ring effect still dominates the particle redistribution process. However, as the temperature rises further, interface-descending rate becomes large enough to form a continuous particle layer (Fig. 3c). The interface capture effect is so strong that the formed islands integrate quickly and cover the whole drop surface. This quasi-solid layer retains until the end of drying and directly deposits at center.

The colors evolution of this layer from red to green further confirms the ordered assembling of particles on surface. Photonic band gap (PBG) of periodic dielectric materials, known as photonic crystal, demonstrates typical structure color^36^,^37^,^38^ due to Bragg diffraction. As the droplet evaporates from spherical cap shape to flat liquid plate, the area of air-liquid interface decreases and causes the compression of the assembled particle (inset in Fig. 3d). Decreasing of particle spacing brings about blue shift of PBG which coincides with the color changes. Owning to the proper particle size, the structure color varies in the visible light region for both convenient observation and detection, which is confirmed by real-time reflection spectrum (Fig. 3d, Fig. S5).

**Figure 4:**
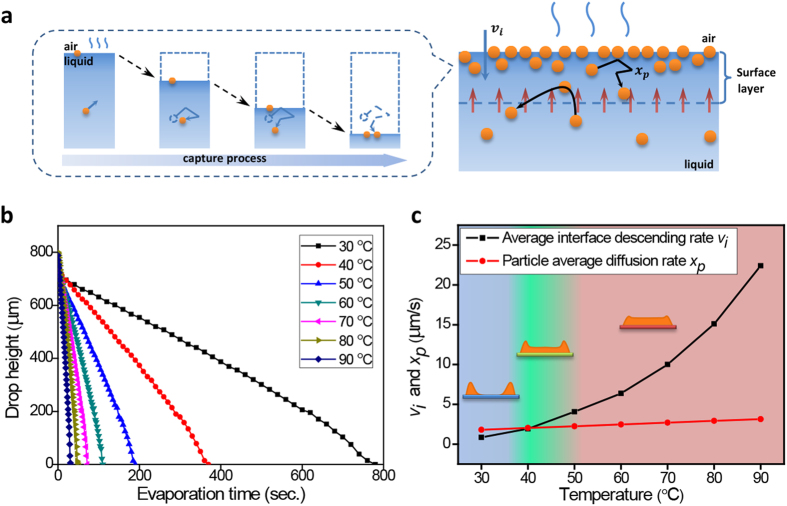
Race between interface descending rate and the corresponding average particle diffusion rate determines the dominance of edge accumulation or surface accumulation. (**a**) Schematic of the rate-dependent interface capture process. (**b**) Drop apex descending evolution at different evaporation temperature. Increasing evaporation temperature accelerates the surface descending rate and decreases the average drying times. (**c**) Phase diagram of the deposition morphology at different evaporation rate. Rapid growth of average interface descending rate *v*_*i*_ and the corresponding particle average diffusion rate *x*_*p*_ declares the dominant field of coffee-ring effect (blue) at low evaporation temperature and surface accumulation (red) at high temperature, green region represent the transition zone of these two process.

Real-time evaporation kinetics records validate this rate-dependent interface capture process. We use video microscopy to quantify the spatio-temporal evaporation profile of the droplet drying at different temperature (inset in Movie S1, S2, and Fig. S6). The race between interface receding and particle diffusion reverses by the acceleration of evaporation, which determines the main direction of particle flow during the drying process (Fig. 4). At low evaporation rate, particles diffuse faster than the descending interface that the air-liquid interface fails to capture these nimble particles. Therefore, they still disperse evenly in the entire drop and finally transport to the contact line by capillary outflow. However, this unbalance situation could be changed by controlling evaporation rate. Increasing evaporation temperature accelerates the surface descending rate and decreases the average drying times (Fig. 4b). According to calculation, the average interface descending rate *v*_*i*_ = *h*/*t*_*f*_ grows faster than the particle average diffusion rate *x*_*p*_ = 2(*Dt*/*π*)^1/2^ (per second) when rising the evaporation temperature. Here *h* is the original height of droplet, *t*_*f*_ the final evaporation time; the diffusion constant *D* is estimated from the Stokes-Einstein relation *D* = *k*_*B*_*T*/6*πηr*, where *k*_*B*_ is Boltzmann’s constant, *T* the temperature, *η* the viscosity of solvent, and *r* the hydrodynamic radius of the particles which approximates to spherical particle radius. When the surface descends faster than the particle diffusion, particles of random walk in vertical evaporation flow will be captured by the drop surface (Fig. 4a), as shown in the simulation process (S7, Mov. S5). In this drop system, the changeover is realized near 40 °C and the interface velocity is large enough to capture the logy particles near surface. When impinge the air-liquid interface, these captured particles will assemble into isolated islands by lateral capillary force^39^. Particle islands appear and then piece together to form continuous layer with continuous evaporation, which consists well with the observation results in Fig. 3. Further temperature rising will accelerate the islands growth, incorporation and thickness growth of solid layer.

According to this model, different drying regimes are simply determined by the evaporation kinetics. The resulting deposition phase diagram shown in Fig. 4b demonstrates the respective dominated field of coffee-ring effect and surface capture effect in different evaporation conditions. At low evaporation rate, particles diffuse faster than the descending interface that most of the particles are transported to edge by capillary outflow, which dominated by coffee-ring effect (blue region). While at rapid evaporation rate, interface descends faster than particles diffusion that the particles are captured at interface forming quasi-solid layer and finally deposit at center, in this region the drying process is dominated by surface capture effect (red region). Between these two regions is the transition zone (green region) that neither of the two effects could be strong enough to dominate the other one, only partial of the particles could assemble on drop surface to form isolate islands or only a few continuous layers. Consequently, the final plate-like deposition is a hybrid result of edge accumulation and surface accumulation. Namely, the ring-like edge still exists but thinner and shorter, and partial particles deposit in center but far from uniform coating.

## Discussion

Different with previous studies that the particles spontaneously aggregate at static interface by reducing the interfacial energy^39^,^40^ or liquid layer confinement^41^, in this regime particles near surface are captured passively by the rapid descending air-liquid interface. The collection of particles on surface actually contains two processes: capture particles from the bulk and fix them on the surface. The former capture process results from the interface capture effect by the kinetically evaporation. The evaporation temperature determines the relative value of interface descending rate and particle diffusion rate, and the race result determines the occurrence of interface capture process. For certain evaporation condition, the capture phenomenon will be easy to realize when the diffusion rate is small, such as in the system with high viscosity or large particle size. The later fixing process is controlled by the particle-interface interaction, which restricts the particle movement at the interface and promotes the particle accumulation. These two capture and fixing processes bring about the surface aggregation phenomenon that overcome the two consumption ways by transportation of capillary outflow and the diffusion from high concentration surface to low concentration bulk. Surfactant was proved to be conducive for fixing the reached particles on drop surface^12^. Suspension with thoroughly cleaned surfactant shows impaired ability to obtain uniform deposition when increasing the evaporation rate (Fig. S8), which is intrinsically different from previous study on surfactant-caused Marangoni flow^5^ and shape-dependent capillary interactions^4^. The weak influence of thermal Marangoni flow on the drying of a sessile droplet^3^,^42^ could be attributed to the following reasons. Firstly, the Marangoni flow is weak in an evaporating water droplet^3^,^29^. Secondly, it is partially counteracted by the enhanced edge aggregation because of the accelerated evaporation due to the hot edge near the heated plate (Figs S2 and S3). Finally, the increased surface viscosity, due to the surface solidification process by particle assembly^4^, also suppresses the surface flow. Therefore, the competition between capillary outflow transportation and surface capture effect is the critical condition that particles finally distribute. Here we control the evaporation rate by changing the drying temperature, other method that influence the evaporation rate such as drying in vacuum shows the similar results (Fig. S9), which also validates this rate-dependent capture process. Additionally, if this capture process is introduced to freestanding droplets like spray drying, hollow spheres structure will form (Fig. S10), which indicates its potential for fabricating hollow structures without template.

In retrospect, inhomogeneous particle distribution from drop center to drop edge results coffee-ring stains. Here the interface capture effect also induces inhomogeneous particle distribution but forms uniform deposition. The difference depends on the destination of the particle accumulation—on the surface or at edge. With well-controlled particle assembling and deposition process in one single droplet, uniform pattern with highly ordered particle assembling can realize at the same time in a very simple and versatile strategy. This would be very significant for fabricating devices based on these uniform patterns with well-ordered assembling as well, such as photonic crystal based sensors and displays^25^,^43^,^44^,^45^. This kinetics-controlled drying regime will not only provide a distinct perspective for drying liquid, such as polymer solution^14^ and blood^46^, but also indicate the direction to enhance the edge accumulation process for fabrication devices with high resolution morphology^13^,^18^ by manipulating the evaporation kinetics.

## Methods

### Particle synthesis

Monodispersed hydrophilic polystyrene nanospheres are synthesized by one-step emulsion polymerization using our modified previous method^24^,^47^. Briefly, St (19.0 g), MMA (1.0 g) and AA (1.0 g) are dispersed in 80 mL water, in which a **tiny** amount of the emulsifier sodium dodecyl benzene sulfonate (SDBS, 0 ~ 0.011 mmol, lower than the critical micelle concentration (CMC)) and buffer agent of ammonium bicarbonate (6.30 mmol) is dissolved previously. Keep the reaction mixture at 70 °C for 0.5 h. Following the addition of initiator for three times, aqueous solution of ammonium peroxodisulfate (APS, 12 mL, 4 mL, 4 mL of 2.12 mmol), keep stirring at 80 °C for 1.5 h, then increasing the temperature to 80 °C before the second addition of APS, and react for another 2 h. Finally, the polymerization carries out at 80 °C for 2 h with continuous stirring for the third addition of APS solution. The resulting latex spheres are used directly without purification. The dispersity of the latex spheres is about 0.005, which is determined by using a ZetaPALS BI-90plus (Brookhaven Instrument) and SEM image. The hydrophobic monodispersed (diameter 255 nm) polystyrene nanospheres are bought from Unisizetech Co. Ltd., China.

### Droplet drying

Water drops containing monodispersed nanospheres (diameter 216 nm) on untreated clean silicon wafer with contact angle about 45° are evaporated in constant temperature & humidity chamber with precise temperature and humidity control—ranging from room temperature to near boiling point of water (*T* = 90 °C) for every ten degrees with constant humidity (50%). We study mass fraction (*ω*) varying from 10^−1^ to 10^−4^ and droplet sizes (*V*) vary from 100 μL to 0.5 μL. Trace amount of surfactant (Sodium Ddecyl Sulfonate, SDS, concentration from 10^−6^ to 10^−4^) is added to investigate the fixation effect of surfactant molecular to colloidal particles on the air-liquid interface. Droplets containing other particles with hydrophobic surface or polydispersed size (diameter 170 nm and 360 nm) are measured at the same condition. The environment humidity when dying on hot plate is controlled with the help of a humidifier.

### Deposition morphology measurement

We use stylus profilometer (MicroFig. Measuring Instrument SURFCORDER, ET-4000, Kosaka Laboratory Ltd) to measure the profile of each deposition obtained from different drying conditions. The profile variation of each deposition formed at different drying conditions indicates the degree of the suppressed coffee-ring effect. The cross section area fraction of the ring-region and center-region in final deposition are measured by software program (MATLAB R2012b, MathWorks). The ordered assembling of the monodispersed nanospheres in deposition are confirmed by SEM image (JEOL S-4800).

### Drying process monitoring

The drying process is recorded via stereomicroscope (Zeiss SteREO Discovery. V8 microscope integrated AxioCam MRc 5 CCD camera) on hot plate (KER3100-08S hot plate, Shanghai Millimeter Precision Instrument Co., Ltd) customized for microscope. Two separate light sources from side view of droplet are used to obtain real three-dimensional visualization stereoscopic images, which could reveal detail information on drop surface. The surface assembling process is observed via optical microscope (Leica DM2500), focusing on region about 1mm at drop apex and tracing the descending surface to acquire snapshots of different drying stages. The air-liquid interface decreasing rate is directly recorded on an OCA20 machine (DataPhysics, Germany) at different substrate temperature and measured by software program (MATLAB R2012b, MathWorks).

### Reflection spectral of drop surface and deposition

Surface reflection spectra and its evolution are detected by Ocean Optics (Dunedin, FL, USA) HR 4000 fiber optic UV−vis spectrometer in reflection mode, with incident light normal to the drop apex. The spectrometer is integrated on a microscope (OLYMPUS BX51) to monitor microdomain reflection spectral on droplet, characterizing the formation of crystal domain shown in Fig. 3. Reflection spectral of deposition is acquired in same method with incident light normal to deposition surface.

### Infrared thermal image of temperature distribution on drop surface

Real time infrared thermal image is acquired by using an IR camera (FLIR A655sc) to investigate the temperature distribution and its evolution on droplet surface. The image is obtained from vertically by drying droplets on silicon with different temperature controlled by hot plate (Torrey Pines Scientific, HS65-2).

## Additional Information

**How to cite this article**: Li, Y. *et al*. Rate-dependent interface capture beyond the coffee-ring effect. *Sci. Rep.* **6**, 24628; doi: 10.1038/srep24628 (2016).

## Supplementary Material

Supplementary Information
