# Erratum: Rate-dependent interface capture beyond the coffee-ring effect

**DOI:** 10.1038/srep27963

**Published:** 2016-06-27

**Authors:** Yanan Li, Qiang Yang, Mingzhu Li, Yanlin Song

Scientific Reports 6: Article number: 24628; 10.1038/srep24628 published online: 04192016; updated: 06272016.

An incorrect Supplementary Information file was inadvertently published with this Article. In addition, Supplementary Movies 1–5 were omitted. The correct Supplementary Information and Supplementary Movies now accompany the Article.

