# Supplementary material for: Rate-dependent interface capture beyond the coffee-ring effect
Source: Sci Rep. 2016 Apr 19;6:24628. doi: 10.1038/srep24628 (PMC4835725; doi:10.1038/srep24628)
Supplement: Supplementary Information [file srep24628-s1.pdf]

Rate-dependent interface capture beyond the coffee-ring effect

Supplementary Information

Yanan Li[1,2], Qiang Yang[1,2], Mingzhu Li[1] & Yanlin Song[1]

[1]Key Laboratory of Green Printing, Institute of Chemistry, Chinese Academy of Sciences (ICCAS), Beijing Engineering Research Center of Nanomaterials for Green Printing Technology, Beijing National Laboratory for Molecular Sciences (BNLMS), Beijing, 100190, P. R. China.

[2]University of Chinese Academy of Sciences, Beijing, 100049, P. R. China.

★E-mail: ylsong@iccas.ac.cn

Content

1. Similar results for particles with hydrophobic surface or polydispersed size 2
2. Pinned contact line confirmed by stereo microscope 3
3. Surface temperature distribution and evolution revealed by infrared thermal imaging 4
4. Suppressed coffee-ring effect on heated substrate 6
5. Ring stains formed at high temperature and high humidity 8
6. Structure color on drop surface originates from the highly ordered assembling of monodispersed particles 9
7. Spatio-temporal evaporation profile of the suspensions drying at different temperature 11
8. Kinetics flow of solvent and solute determines the particle concentration on surface layer 12
9. Computation simulation of the drying process at different evaporation condition 13
10. Trace surfactant helps to fix the collected particles at air-liquid interface 14
11. Similar results of suppressed coffee-ring effect achieved from drying in vacuum analogous to evaporation temperature control 15
12. Hollow structure fabricated by this interface capture effect in the situation of free standing fog droplets 16
13. Influence of particle concentration, solvent composition and substrate wettability 17

References 18

1. Similar results for particles with hydrophobic surface or polydispersed size

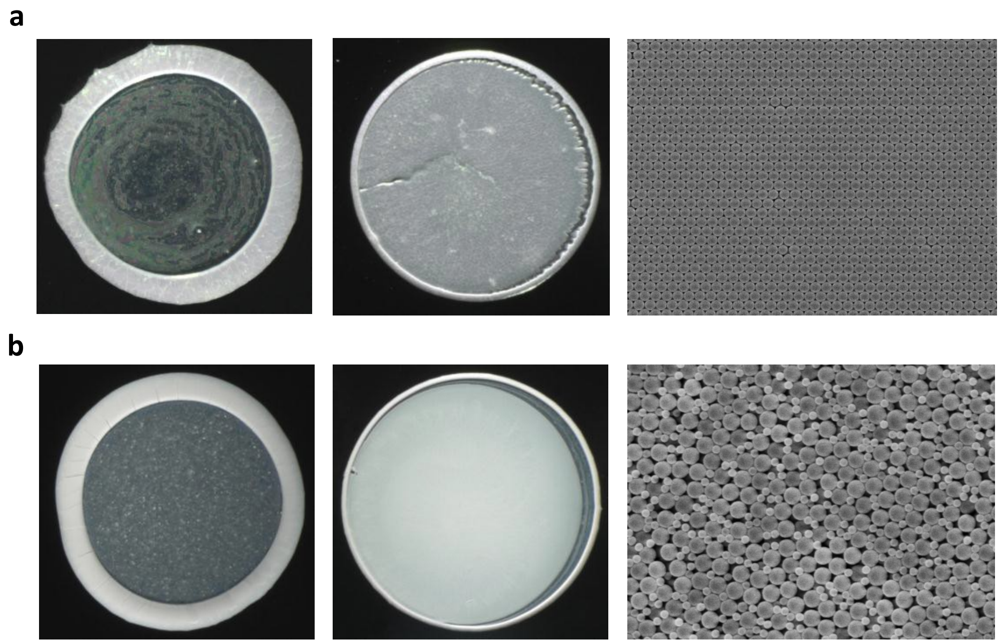

Figure S1. Both monodispersed nanospheres with hydrophobic surface and polydispersed nanospheres with hydrophilic surface show suppressed coffee-ring effect when drying at rapid evaporation rate. a, Final deposition of hydrophobic particles: ring-stain at low evaporation rate (left) and uniform deposition at rapid evaporation rate (middle). **b,** Final deposition of polydispersed nanospheres: ring-stain at low evaporation rate (left) and uniform deposition at rapid evaporation rate (middle). Monodispersed size also results ordered assembling (**a** right), while polydispersed size results disordered assembling (**b** right). The scale bar of optical image is 0.5 mm and SEM image is 1 μm.

Deposition of hydrophobic particles is obtained by drying 5 μL aqueous droplet suspended hydrophobic polystyrene nanospheres with diameter 255 nm on untreated clean silicon wafer. Deposition of polydispersed nanospheres is obtained by drying 5 μL aqueous droplet suspended core-shell hydrophilic P(ST-MMA-AA) nanospheres with diameter ranging from 170 nm to 360 nm on silicon wafer (mixing particle with diameter of 170 nm and 360 nm by mole ration of 1:1). These similar results analogous to preceding used monodispersed hydrophilic nanospheres excludes the possibility that surface accumulation process is dominated by interface energy controlled interface adsorption or particle size distribution, but the results of interface capture caused by kinetic evaporation.

2. Pinned contact line confirmed by stereo microscope

Movie. S1. Droplet with slow evaporation rate produces obvious coffee-ring effect. A drop of suspension (5 μL in volume, 3.6 mm in diameter, $\emptyset$=2% wt in water) drying on untreated clean silicon substrate with low substrate temperature ($TT = 30℃$), recorded by stereo microscope. Side view (inset) recorded by video microscope indicates the contact angle evolution and confirms the pinned contact line, fifty times playback speed.

Movie. S2. Droplet with rapid evaporation rate shows suppressed coffee-ring effect. A drop of suspension (5 μL in volume, 3.6 mm in diameter, $\emptyset$=2% wt in water) drying on untreated clean silicon substrate with low substrate temperature ($TT = 70℃$), recorded by stereo microscope and playback at five times speed.

3. Surface temperature distribution and evolution revealed by infrared thermal imaging

Movie S3. Infrared thermal imaging of the droplet drying at low temperature. Infrared thermal evolution shows gentle surface temperature gradient when the drop dries on substrate with low temperature (5 µL in volume, 3.6 mm in diameter, $\emptyset$=2% wt in water, untreated clean silicon wafer with temperature $TT = 30$℃)

Movie S4. Infrared thermal imaging of the droplet drying at high temperature. Infrared thermal evolution shows steep surface temperature gradient when the drop dries on substrate with high temperature (5 µL in volume, 3.6 mm in diameter, $\emptyset$=2% wt in water, untreated clean silicon wafer with temperature $TT = 60$℃)

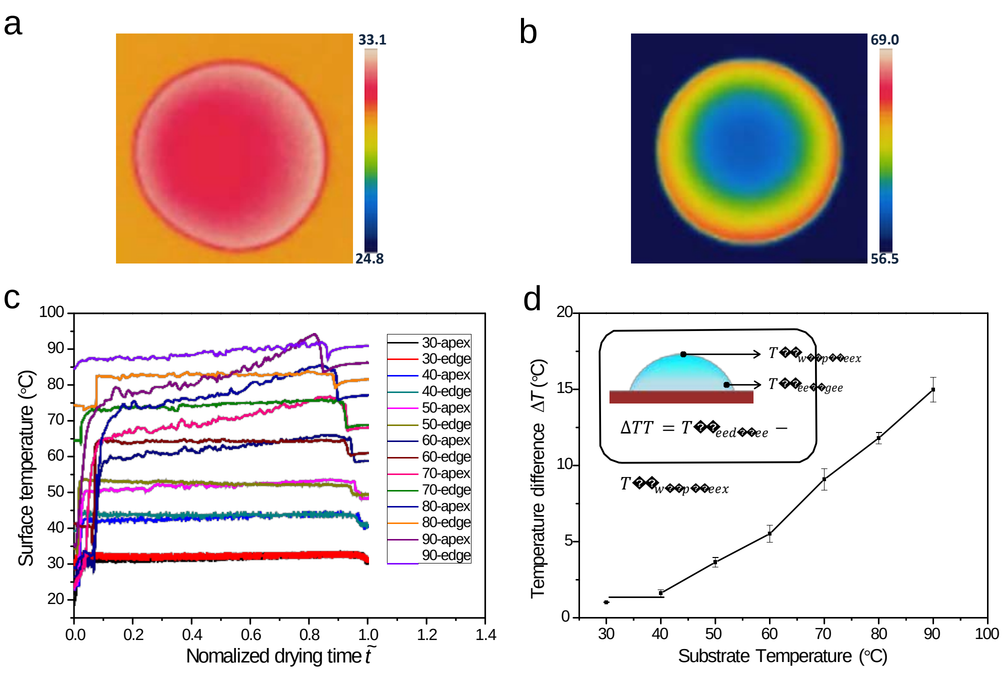

Figure S2. Enlarged temperature difference between drop edge and apex by substrate heating. a, b. Infra images of temperature distribution on drop surface reveal that surface temperature gradient at high temperature (**b**) is more distinct than the one drying at low temperature (**a**). **c.** Surface temperature evolution of a droplet on different substrate temperature; **d,** The initial temperature gradient on a droplet increases as arising the substrate temperature.

Figure S2 demonstrate the enlarged temperature difference between drop edge and apex by heating substrate. The temperature at drop edge is higher than that at the drop apex because of the direct contact with the hot substrate and vertical heat conduct hysteresis. On one hand, the existence of the temperature gradient will cause surface tension gradient on the air-liquid interface inducing thermal Marangoni flow to suppress the coffee-ring effect,[1-5] while on the other hand, high temperature at drop edge will accelerate the edge evaporation, which will enhance the coffee-ring effect. Therefore, the actual influence of the heat substrate to the edge deposition from the view of thermal Marangoni flow and capillary flow need further investigation.

It is worth noting that the infrared image and corresponding temperature profile in Figure S2 was acquired from a rough measurement without precise calibration. As the camera receives radiation from not only the object itself, but also collects radiation from the surroundings reflected via the object surface when viewing an object. Both these radiation contributions become attenuated to some extent by the atmosphere in the measurement path. The temperature provided by the camera is not the real fluid temperature but an averaged temperature of a column going from the substrate to the camera.[6] To acquire accurate surface temperature distribution, precise infrared properties (emissivity, reflectivity, and transmittivity) of the

emulsion needs carefully measured, and the substrate radiation and surface reflection should be manipulated carefully.

As the deduction process based on the infrared data relies on the tendency rather than the precise value of the temperature difference between drop edge and drop apex, the conclusion remains valid. Though the increased temperature could induce thermal Marangoni flow, the actual influence of this thermal Marangoni flow was proved to be tiny according to the following contrast experiments (Figure S3, S4) because it is suppressed by enhanced edge evaporation caused edge aggregation and the increased surface viscosity due to the surface solidification process by particle assembly.

4. Suppressed coffee-ring effect on heated substrate

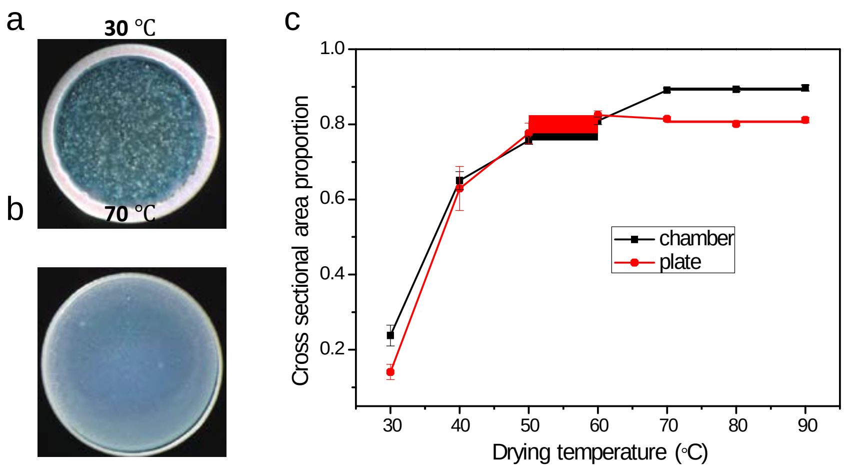

Figure S3. The suppressed coffee-ring effect on heated substrate. a, b, Images of the final deposition morphology of droplet drying on substrate with low temperature (**a,** $TT = 30$℃) and high temperature (**b,** $TT = 70$℃). Ring-like

fashion

forms at low substrate temperature and uniform deposition is formed at high temperature. **c,** Quantified coffee-ring effect by the cross section area proportion of ring region in the deposition pattern acquired from drying on hot plate consists on the whole with that in constant temperature & humidity chamber except little deviation at high temperature region. The cross section area proportion of ring region decreases as the center region arises when the chamber temperature increasing, which indicates the gradually suppression of coffee-ring effect. Depositions are uniform with less ring-region at high temperature than that on hot plate at the same drying temperature, because the evaporation rate gradient is enlarged by heat transfer hysteresis on hot plate. The scale bar, 0.5 mm.

As it is difficult to direct observe the drying process in a constant temperature & humidity chamber (CTHC, a facility with well-sealed space for precise temperature and humidity control), we carry all the optical experiments on a heat plate with precise temperature control at the similar environment humidity as well. In order to exclude the deviation may cause by different heating methods; we compare the drying results from these two heating facilities by deposition morphology and infrared thermal analysis. What is interesting is that the whole morphology variation tendency consists well with that in CTHC except a little deviation at high temperature (Figure S3, c). More intriguing is that the deposition is even more uniform by drying in CTHC than that on a hot plate in high temperature. As the surface temperature gradient, which is believed to induce thermal Marangoni flow that repels the coffee-ring effect[1-5], is larger on a hot plate than that in a CTHC because of the heat hysteresis[2] between drop bottom and apex (Figure S2), the deposition on hot plate should have been more uniform. The results here indicates that the influence of temperature gradient caused Marangoni flow in this system seems to be much weak than expected. Actually, the influence of substrate temperature to the deposition morphology has been controversial for years. Several opposite results contrary to the temperature-driven Marangoni flows[7-10] shows the Marangoni flow with opposite direction[10] or its absence of in water[11], and enhanced edge evaporation because of the high temperature at drop edge close to the heated substrate promotes the edge accumulation rather than the opposite Marangoni flow. This deposition morphology variation from CTHC and hot plate seems to support the latter as well. Heat transfer hysteresis between drop bottom and apex, which can be negligible in CTHC because of the more uniform heat transfer, induces the temperature gradient on drop surface and rapid evaporation at drop edge accelerates the edge accumulation of particles. The temperature difference enlarged by increasing the substrate temperature (Figure S2) will enhance this nonuniform deposition process. The mentioned weak flow intensity in water-based emulsion is not the only reason for the

absence influence from thermal Marangoni flow on the drying of a sessile droplet. Theoretically, high temperature at drop

edge will drive liquid to the cold region, i.e., drop apex, because of the surface tension is usually lower at high temperature, which seems to suppress the edge aggregation process. However, this suppression effect could also be counteracted by the accelerated edge evaporation due to the warm edge near the hot plate (Fig. S2, S3).[7] The final influence of substrate temperature depends on the relative intensity of these two processes. Therefore, though we cannot quantitatively tell the weighting factor of the influence from the enlarged surface temperature, induced thermal Marangoni flow or enhanced edge evaporation, its actual influence to the deposition formation shows to be small in water drop system based on both the previous study and our control experiments. Latterly, we will reveal that the suppressed coffee-ring effect mainly origins from the rate-dependent surface capture effect. The competition between capillary outflow transportation and surface capture effect is the critical condition that particles finally distribute. Moreover, the increased surface viscosity due to the surface solidification process by particle assembly[12] could also dramatically suppress the surface flow that might be caused by temperature-induced surface tension gradient. The detail quantitative effect of thermal Marangoni flow on the drying of a sessile droplet needs further study when considering the particle-particle interaction and particle-solvent molecule interaction to improve the classical hydrodynamic theory.

5. Ring stains formed at high temperature and high humidity

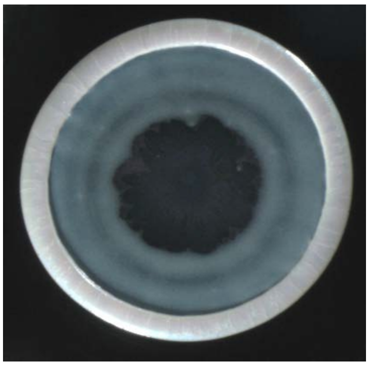

Figure S4. Droplet drying at high humidity (100%) and minimum air convection in CTHC shows distinct ring-like deposition because of the slow evaporation rate. The scale bar is 0.5 mm.

Environment relative humidity of experiments on hot plate is controlled near 50%, approximately consistent with that in CTHC. Water evaporates slowly at high humidity—a kinetic process of water molecular from liquid phase to gaseous phase. As we expected, droplet drying at high humidity (100%) and minimum cross-ventilation in CTHC shows distinct ring-like deposition because of the slow evaporation rate. Once again, this result proves the reason of interface capture effect that it is rate rather than temperature that matters.

6. Structure color on drop surface originates from the highly ordered assembling of monodispersed particles

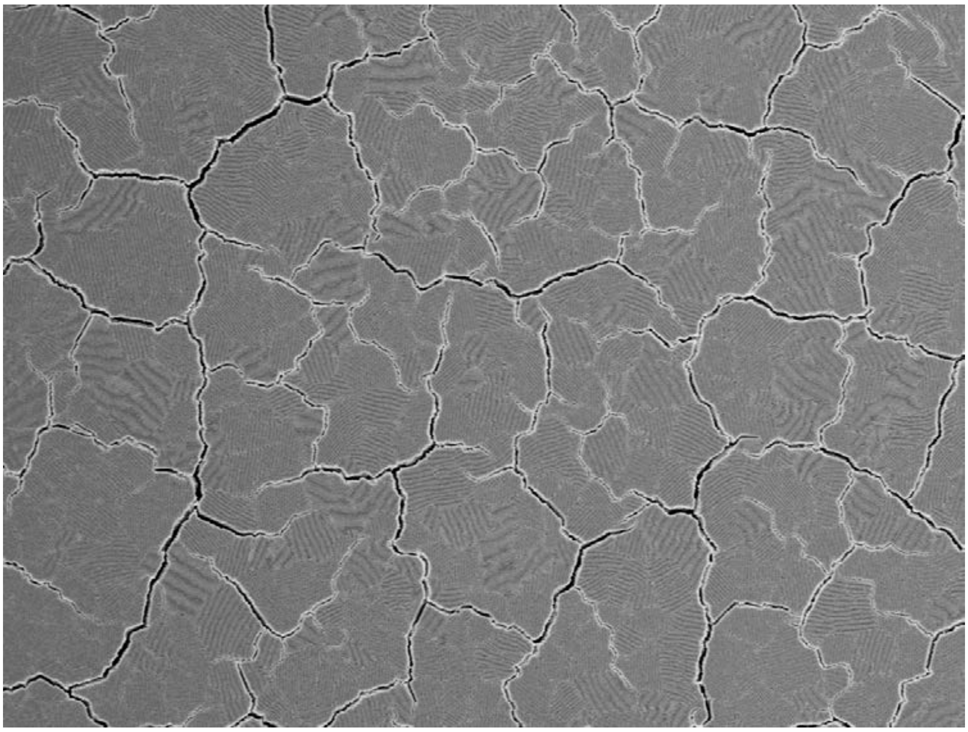

Figure S5. SEM image of the formed micro domains indicates the multi-site nucleation growth process. Ordered assembling of particles in deposition of droplet drying at 75℃. The cracks between the micro domains origin from the stress-release process after the replacement of water by air at the final stage of drying (scale bar, 20 μm).

The peak evolution reveals the growth of surface assembling process, from nucleation to island growth to solid-layer formation. At the beginning (stage 0 and 1 in Figure 3, **d**), nucleation and a little isolate islands are too small to show distinct diffraction to incident light that the reflection spectrum originates from the bulk suspension—no peak appears. As the islands growing up, light diffraction by the ordered assembling of particles on surface becomes more and more obvious that a little peak comes into being (stage 2, peak at 636 nm). With continuous evaporation of the droplet, the islands integrate together and form a quasi-solid layer, thus the diffraction to incident light is strong enough that the intensity of the peak increases. Additionally, with the descending air-liquid interface, the assembled particles are compacted as the surface area shrinks that brings about the blue shift of reflection peak (stage 2-6, peak position shifts from 636 nm to 567 nm).

Introduction to PBG of PC:

Photonic crystals are periodic optical nanostructures that affect the motion of photons in much the same way that ionic lattices affect electrons in solids. Highly ordered assembling of particles in nanometer or micrometer scale has the similar band-gap to photons by Bragg diffraction[13-17]. A simple approximate relationship between bandgap position and interplanar crystal spacing is demonstrated by equation[18-21]:

$mmmm = 2D$���n��^{2} − s��s_e��n��^{2} (θ��)

Where mm is an integer determined by the order given,

mm is the wavelength of incident wave,

DD is the spacing between the planes in the atomic lattice, for closed-packed face centered cubic crystal from [111] face $DD =$ �$\frac{2}{3}$ d��, where d is the diameter of the particle,

n_{eff} is the effective refractive index demonstrated by $n_{eff}^2 = n_p^2 f + n_s^2 (1-f)$, where n_p and n_s is the refractive index of particle and space medium respectively, f is the packing factor, θ is the angle between the incident ray and the scattering planes.

From the equation, the change of particle space will influence the interplanar crystal spacing for the same colloid system. The compression caused by drop surface shrinkage decreases the particle space, which brings about the blue shift of band gap. Additionally, in the final stage that water in particle space will be replaced by air after the quasi-solid layer completely dry. This replacement from high refractive index space medium ($n_{water} = 1.33$) to low refractive index space medium ($n_{air} \approx 1$) will increase the effective refractive index n_{eff}, which also brings about the blue shift of band gap. Additionally, the position of the photonic band gap significantly depends on the particle size *d*, therefor by selecting proper particle size, we could ensure the whole position shift of band gap (corresponding to the color change of the particle layer) during the compression process varies in the visible light range for convenient observation. The variation of the particle interspace indicates that the assembling of particles on water surface is far from close-packed but filled with solvent layer. Continuous evaporation of the solvent results the replacement of water by air, which often cause cracks in the final deposition (Figure S6).

7. Spatio-temporal evaporation profile of the suspensions drying at different temperature

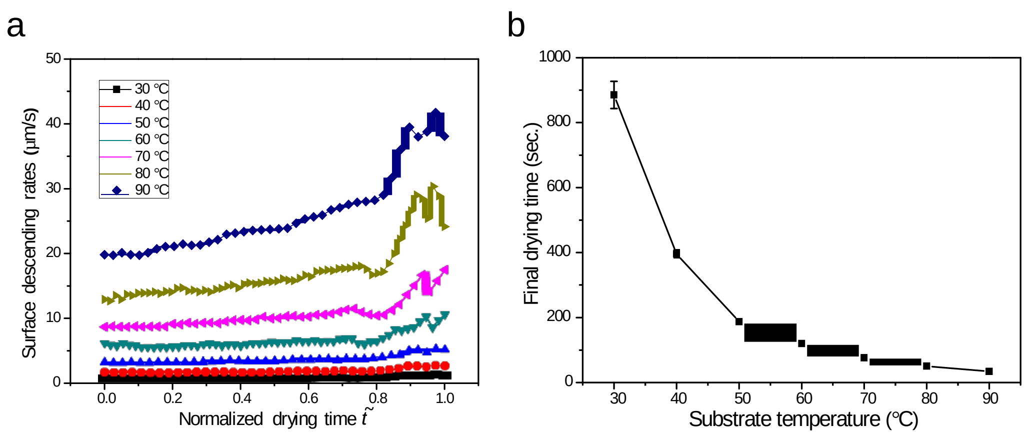

Figure S6. Real time drop height descending rate at different substrate temperature. Increasing evaporation temperature accelerates the surface descending rate with decreasing of average drying time (inset). Additionally, the descending rate shows approximate constant at low temperature and slightly accelerates at the final stage of drying, which is especially distinct at the high temperature.

The air-liquid interface shrinkage process is directly recorded on an OCA20 machine (DataPhysics, Germany) from side view. By monitoring the height profile evolution, we trace the height descending curves and calculate the descending rates of drop surface via software program (MATLAB R2012b, Math Works). Different from previous studies that evaporation will slow down because the coverage of particles on surface results decreasing of effective evaporation area[22], our experiment demonstrates that drop apex descends accelerate at the final stage of drying. The acceleration of apex shrinkage at the final stage attributes to the temperature gradient on drop surface becomes flat on the influence of heat transfer (See Figure S4), the primary relative cold drop top becomes as worm as drop edge near substrate, thus accelerates the evaporation. Because the average particle diffusion distance is calculated by statistical approach, we use average interface descending rate rather than real time drop height descending rate to deduce the condition of interface interception. Moreover, average rate could be more representative of the whole evaporation process for the reason that the deposition forms after the drop completely drying.

Deviation between theory calculation and experiment observation may stems from simplified conditions. Firstly, we took the temperature distribution to be uniform in the whole droplet which actually proved to be nonuniform by infra image in Figure S2. Additionally, temperature gradient may induce surface tension and viscosity distribution, which influence the particle diffusion velocity. Secondly, average interface descending rate is used to deduce the condition of interface capture. Real time drop height descending rate revealed that the descending rate keeps constant at low temperature and slightly accelerates at the final stage of drying, especially at the high temperature. Thirdly, room humidity may vary when observing the surface assembling process, which may influence the instantaneous evaporation velocity. Fourthly, the diffusion model obtained from statistic derivation of random walk theory determines the particles occurred at air-liquid interface can be random. The usage of average diffusion rate excludes the occurrence of particles at interface that above or below average scale. Well actually, for those deviating from average level should occur at interface. Though these factors may influence the result of interface capture, only the critical temperature at which interception occurs may vary in a small limited region. The tendency that increasing the evaporation temperature brings about interface interception remains valid. Therefore, the boundary between coffee-ring effect and interface capture effect in phase diagram shown in Fig. 4b is not definite, just as the blurry boundary between smooth and ruggedness of the deposition morphology.

8. Kinetics flow of solvent and solute determines the particle concentration on surface layer

We demonstrate this mechanism by the race of interface receding velocity and average particle diffusion distance. In the reference frame of interface, the formation of the surface quasi-solid layer can also be explained by mass conservation in a tiny region near the surface, including solvent and particle exchange. In this tiny layer, evaporated solvent molecules could be replenished from the liquid phase below, and particle accumulation at interface by capture effect could also be consumed by desorption and diffusion. The balance of this mass conservation depends on the kinetics of each flux. When the condensed particles are neither depleted by desorption nor diluted by replenishing bulk flow with relative lower concentration, the average particle concentration in the tiny region will increase. Finally the liquid surface will gradually be replaced by a particle layer. We image a thin layer on liquid surface, in which solute and solvent exchange between air and bulk will determine the formation of surface solidification. Solvent evaporation flux f_e (blue evaporation streamline in Fig. 4a) and vertical flow (red arrow in Fig. 4a) f_v determine the quantity of solvent, and particle diffusion will influence the quantity of solute (nanospheres) in the thin layer. Both the flow of solvent and solute will determine the concentration of particles in the thin layer. On the side of solvent, If vertical solvent flow cannot replenish the solvent loss due to reasons like viscosity or other factors that weaken the mobility of solvent, particles in the layer will be concentrated by the net loss of solvent. On the side of particles, with the evaporation of solvent, the retained particles and vertical flow, carrying particle into the layer will increase the particle content in the layer. The only particle loss by diffusion is limited by particle average diffusion distance[23]

$$x_{p} = \sqrt{2 D \,[illegible] / \pi}$$

The particle diffusion constant D is estimated from the Stokes–Einstein relation

$$D = k_B T / 6\pi\pi\pi [illegible],$$

where k_B is Boltzmann's constant, T the temperature, π the viscosity of water which varies with temperature, and π[illegible] the hydrodynamic radius of the particles which approximates to particle radius. In summary, if the condensation of particle near surface by solvent evaporation and vertical replenishment cannot be suppressed by the diffusion consumption, the net accumulation will bring about particle stacking near surface—the beginning of surface solidification.

This mass exchange model is conducive to explain the ambient particle assembling shown in Fig 3b, as which may be confused that particle should have appeared in the drop apex region preferentially because of the larger descending rate in the drop apex region—due to the geometrical spherical cap, the absolute descending distance in drop apex is larger than other position at the same time. As shown in Fig. 2d, increasing the evaporation rate does not eliminate the capillary outflow but changes the relative proportion of particle flow by the capillary outflow. Therefore, in the reference of air-liquid interface, the capillary outflow acts as replenishing bulk flow, which is absence in the vertical direction to drop apex. This process promotes the particle impinging the air-liquid interface and results the preferential appearance of ambient particle assembling.

From this kinetics perspective, this model will be helpful to understand other phenomenon involving surface solidification such as crust effect in polymer solution[22,24] and blood[25,26], in which the surface of liquid with large viscosity can solidify quickly with little descending distance. The low viscosity drop system in our experiment can also be explained by this mass exchange mode. Increasing the evaporation temperature will dramatically enhance the evaporation flux f_e from liquid to air. Meanwhile, the particle diffusion rate is not much sensitive to that of surface condensation according to our calculation. Therefore, particle near surface can accumulate by the net input flow.

Table S1.Viscosity of water at different temperature[27]:

T [°C]	30	40	50	60	70	80	90
η_{H_2O} [10^{-5} Pa • s]	80.07	65.60	54.94	46.88	40.61	35.65	31.65

9. Computation simulation of the drying process at different evaporation condition

Movie S5. Computer Simulated drying process of a single droplet evaporating at different temperature. Ring stains formed at low evaporation temperature (top, $TT = 30$°C) due to the dominated radical capillary out flow. While a relative uniform deposition formed at high evaporation temperature (bottom, $TT = 70$°C) because of the dominated interface capture process.

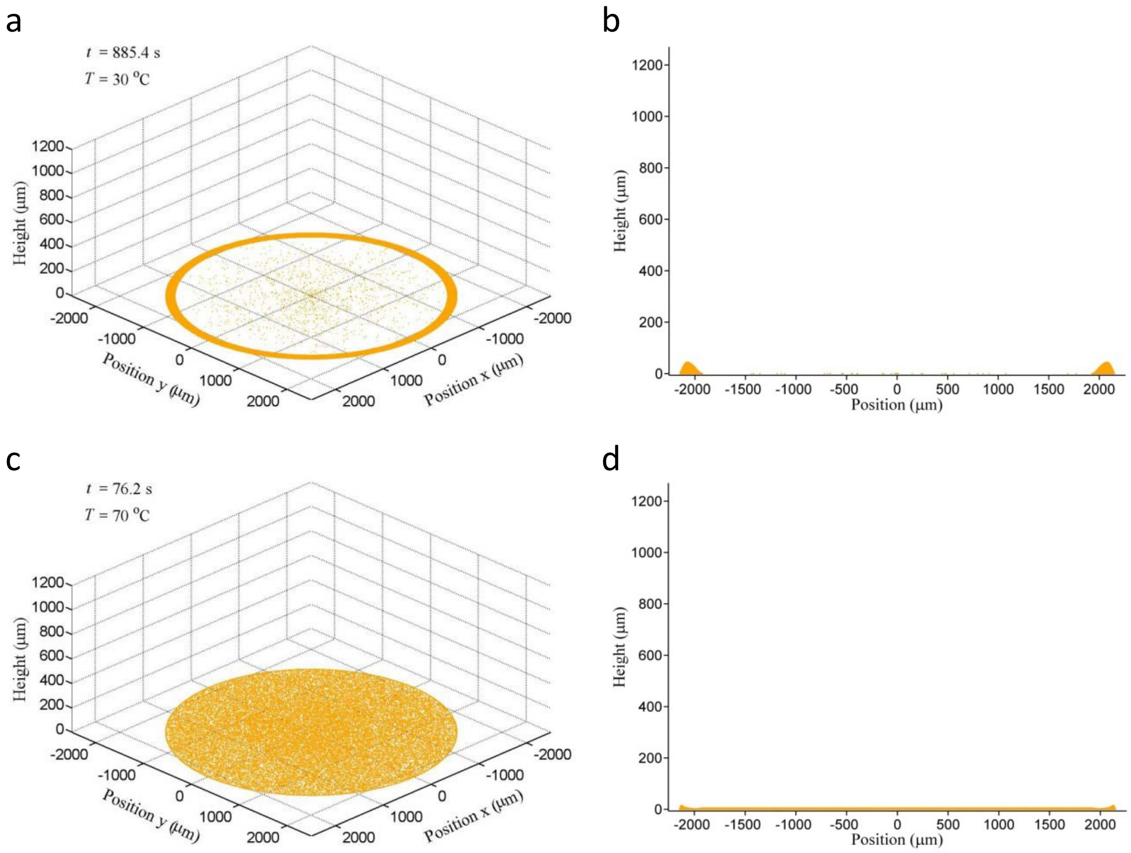

Figure S7. Computation simulation of the drying process of a single droplet at different evaporating temperature. a, b. Front view and side view of the simulated drying process of the droplet at low temperature ($TT = 30$°C). **c, d.** Front view and side view of the simulated drying process of the droplet at relative high temperature ($TT = 70$°C).

The simulation is realized via the finite element modeling with MatLab by introducing the particle-interface interaction and particle-solvent interaction in previous model[28] with neglecting the Marangoni flow and particle-particle interaction due to their small influence to the particle redistribution in this water drop system as we discussed above (Fig. S2-4).

10. Trace surfactant helps to fix the collected particles at air-liquid interface

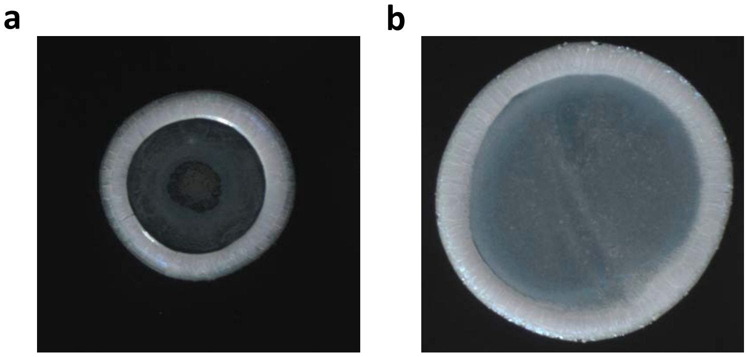

Figure S8. For solution with thoroughly cleaned of residual surfactant, increasing the evaporation rate shows weaken ability to suppress the coffee-ring effect. a, Ring stains still exists when drying at slow rate though the contact line depins during the dying process for lack of surfactant ($TT = 30$°C). **b,** With the absence of surfactant, ring-like edge is obvious even when the drop drying at high temperature ($TT = 70$°C), and only partial particles deposits at center. Even so, the ratio of center region is larger than that of low drying temperature.

The residual surfactant is thoroughly cleaned by centrifugation for ten times (14800 r/min for 30 minutes).

According to this capture mechanism, the accumulation of particles on surface contains two processes: transportation of particles from drop inner to drop surface, and the fixation of particles on air-liquid surface during the drying process. The magnitude of the former depends on the race of interface collection and particle diffusion, while the latter is significantly influenced by the capillary interaction between particles and air-liquid interface. Suspension with residual surfactant thoroughly cleaned shows impaired ability to obtain uniform deposition when increasing the evaporation rate (Fig. S9 b). Ring stain still appears at low evaporation temperature with contraction of the three-phase contact line (Fig. S9 a) for lack the adsorption of micelles onto colloid and substrate surfaces[29]. Adding a SDS weight fraction as small as 1×10^{-5} once again promoted kinetically induced quasi-solid layer growth at the liquid–air interface. Colorful surface luster reappears on drop surface quickly compared with that appears only at a small annulus region near edge during the late stage of drying.

However, it should be noted that the function of surfactant in the mechanism of interface capture is intrinsically different from that in previous studies[29-31] on suppression of coffee-ring effect. The mechanism of added surfactant (concentration scale about 10^{-3} in weight percent) in previous studies to suppress the coffee-ring effect stems from the Marangoni flow caused by the surfactant concentration gradient on the drop surface during the drying process. While in this capture model, trace amount of (~10^{-5}, not only far below the critical micelle concentration of the surfactant but also much dilute than the concentration that could inducing Marangoni flow[30]) surfactant molecules which typically already exists in the fresh fabricated nanoparticle emulsion, covering the air-liquid interface induces a short ranged dipole-dipole interaction[32] and strengthen the captured particles on surface[33-35]. When solution with thoroughly cleaned of residual surfactant drying at high temperature, even though the interface descends fast than diffusion and also the particle could reach the drop surface, the weak interaction between particle and interface fails to fasten the particles from desorbing into bulk. However, it is also noteworthy that the existence of surfactant is conducive to this capture process but indispensable. For the first reason is that more particles still deposit at center than that drying at low evaporation temperature though the ring-like edge is obvious, which indicates that there are still particles captured and deposit in center though the amount is small. The second is that coffee ring effect is still prominent at the existence of this trace surfactant when drying at slow evaporation rate. Concern that these trace surfactants may cause suppressed coffee-ring effect can thus be dismissed.

11. Similar results of suppressed coffee-ring effect achieved from drying in vacuum analogous to evaporation temperature control

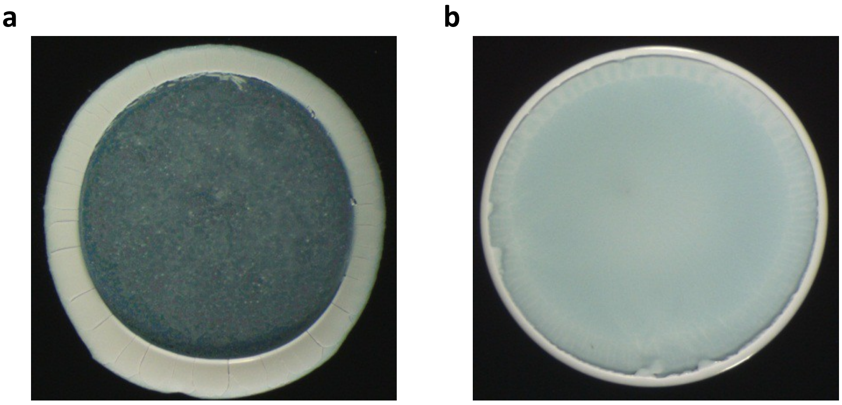

Figure S9. Analogous to evaporation temperature controlled deposition, droplet drying in vacuum shows similar results—suppression of coffee-ring effect in vacuum. a, Deposition formed in normal pressure and room temperature shows distinct coffee-ring stain. **b,** Deposition formed when droplet drying in vacuum but still at room temperature shows suppressed coffee-ring effect, most of the particles deposits at center and only a small part of particles accumulation at contact line. The scale bar is 0.5 mm.

Drying in vacuum could dramatically increase the evaporation rate without increasing the temperature. 5 μL aqueous droplet suspended core-shell hydrophilic polystyrene nanospheres with diameter ranging from 170 nm to 360 nm dries at vacuum degree of 0.1 atm on silicon wafer. This result not only confirms the rate controlled deposition mechanism, but also provides a significant way for samples which are sensitive to temperature, such as biomaterials[36].

12. Hollow structure fabricated by this interface capture effect in the situation of free standing fog droplets

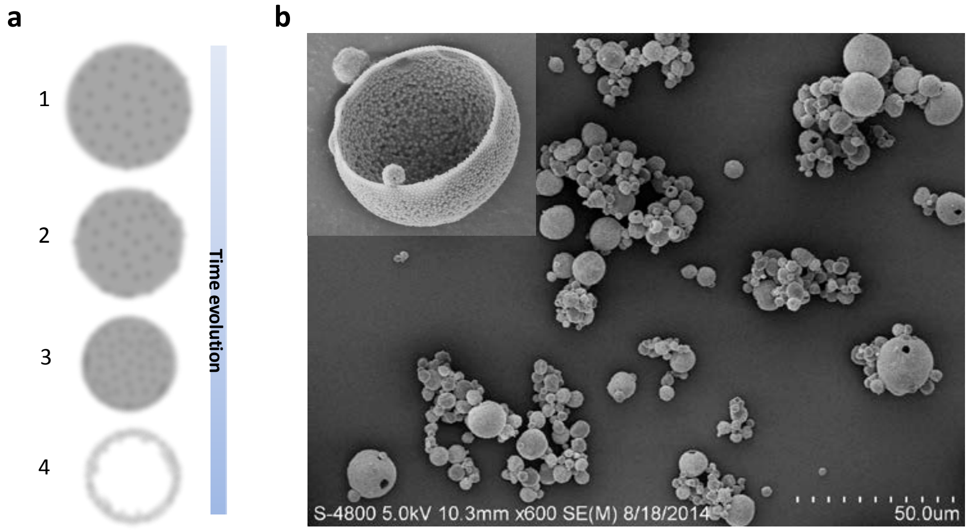

Figure S10. Hollow sphere obtained from spray drying at high temperature. a, Schematic of the formation of hollow sphere by this interface capture process. **b,** Micrometer size fog droplet in a chamber with high temperature (200℃) dries fast and forms hollow sphere (inset, scale bar 2 μm) due to the interface capture effect.

We study the drying droplet on a substrate with spherical cap, and the formed quasi-solid layer only cover the air-liquid interface with partial spherical cap. If this interface capture effect occurs in freestanding spherical drop rather than droplet with partial spherical cap on a substrate, suspended particles will accumulate at the entire air-liquid interface forming a quasi-solid spherical shell during the shrinkage of the drop. Once the thickness and strength of this shell is strong enough, the drop will stop shrinking and the radius of the shell will keep constant. At the same time, liquid suspension inner the shell will continue drying. With the escaping of solvent molecules from the particle interspace in the shell, the space of the solvent inside the shell will be vacant and leaving of residual suspended particles inside the shell, thus forming a hollow sphere structure (Figure S11. a). In this assumption, we drying micrometer size fog droplet in a chamber with high temperature (200℃) dries fast and successfully forms hollow spheres (Figure S11. b). This interface capture effect will inspire new methods on fabricating hollow structures without templates[37], such as hollow spheres and tubes[38]. Different size and thickness can be obtained by manipulating the concentration of the suspension, solvent composition and drying temperature.

Fabrication method: Lab spray dryer (YC-015, Shanghai Pilotech Instrument & Equipment Co., Ltd) is used to obtain this hollow structure. Suspension with concentration of 36%wt is sprayed by nebulizer and carried by heated air with temperature of 200℃ into tubular furnace. The final dried powder is collected by cyclone separator for characterization.

13. Influence of particle concentration, solvent composition and substrate wettability

According to this diffusion model, other factors, such as particle concentration, solvent composition and substrate surface energy could actually influence the final deposition morphology. We find that in ultra-low particle concentration region (weight percent $\omega < 10^{-4}$), the final deposition tends to be ring stains and demonstrates typical cellular flow patterns caused by three-dimensional Rayleigh-Bénard convection[39] at high substrate temperature. Because there are not enough particles to form an entire solid layer, the islands occurred at air-liquid interface are finally desorbed into bulk with the consumption of diffusion and radical outflow at later stage of evaporation. Meanwhile, the high temperature difference between drop edge and drop apex while promote the edge evaporation, which leads obvious edge accumulation. While in the ultra-high region (weight percent $\omega \geq 1.5 \times 10^{-1}$), though surface accumulation still emerges that the deposition becomes uneven with ring-edge broadening and a little higher than center. Because the formed surface solid layer becomes rigid when its thickness is too large to deform, that the surface descends slowly and the residual particles tends to accumulate in the radical direction, similar to the hollow structure formation in Figure S11. This deduction can explain previous studies of reverse results.[7,40]

Choosing highly volatile solvent such as toluene[41] will be analogous to increased evaporation temperature. Its rapid evaporation helps particles to accumulation at air-liquid surface. The system viscosity is also an important factor to this capture process according to the diffusion equation. High system viscosity, such as some polymer solution droplet[7], will significantly suppress the diffusion length of the particles, which would promote the surface solidification according to the mass exchange model we mentioned in Figure S5. In this situation, surface solidification can appear early at relative low temperature. When increasing the evaporation temperature, the formed quasi-solid layer becomes rigid that the interface descends slowly. Further evaporation of solvent will brings about the sinking of the surface quasi-solid layer when losing the support of the under-surface liquid. Surface layer near the drop apex will firstly sink and descend longer distance than that near the edge because of the geometrical shape of droplet, resulting deposition with concave morphology. Therefore, for solution with high viscosity suspending the formation and rigidity of the surface layer will be conducive to obtaining uniform coating. Though this seems to be contrary to our result that high evaporation temperature results uniform deposition, it actually does not contradict the model. Because of the high viscosity, the surface solidification temperature is lower than the system with low viscosity. For low viscosity system, the surface solidification temperature is relative high (about 40℃ in our PS nanospheres-water system), increasing the evaporation temperature will just promote the thickness growth of the quasi-solid layer, which will have deposited on the substrate before it is rigid enough to self-standing. While for the high viscosity system, the surface solidification might appear before the evaporation temperature increase to 40℃, further increasing the temperature will increase the thickness and the strength of the quasi-solid layer, thus forms concave morphology.

Rapid evaporation at hydrophobic surface, especially for super hydrophobic surface, by analogy to free standing sphere drop tends to form hollow sphere (Figure S11), which also means uneven deposition.

References

1. Girard, F., Antoni, M. & Sefiane, K. On the effect of Marangoni flow on evaporation rates of heated water drops. *Langmuir* **24**, 9207-9210 (2008).
2. Ristenpart, W. D., Kim, P. G., Domingues, C., Wan, J. & Stone, H. A. Influence of substrate conductivity on circulation reversal in evaporating drops. *Phys. Rev. Lett.* **99**, 234502 (2007).
3. Lu, G., Duan, Y. Y., Wang, X. D. & Lee, D. J. Internal flow in evaporating droplet on heated solid surface. *Int. J. Heat Mass Tran.* **54**, 4437-4447 (2011).
4. Girard, F., Antoni, M., Faure, S. & Steinchen, A. Evaporation and Marangoni driven convection in small heated water droplets. *Langmuir* **22**, 11085-11091 (2006).
5. Li, Y., Lv, C., Li, Z., Quere, D. & Zheng, Q. From coffee rings to coffee eyes. *Soft Matter* **11**, 4669-4673 (2015).
6. Brutin, D. in *Droplet Wetting and Evaporation From Pure to Complex Fluids* Vol. 1 Ch. 15, 193 (Elsevier, 2015).
7. Soltman, D. & Subramanian, V. Inkjet-printed line morphologies and temperature control of the coffee ring effect. *Langmuir* **24**, 2224-2231 (2008).
8. Kim, J. H., Park, S. B., Kim, J. H. & Zin, W. C. Polymer Transports Inside Evaporating Water Droplets at Various Substrate Temperatures. *J. Phys. Chem. C* **115**, 15375-15383 (2011).
9. Parsa, M., Harmand, S., Sefiane, K., Bigerelle, M. & Deltombe, R. Effect of substrate temperature on pattern formation of nanoparticles from volatile drops. *Langmuir* **31**, 3354-3367 (2015).
10. Park, J. & Moon, J. Control of colloidal particle deposit patterns within picoliter droplets ejected by ink-jet printing. *Langmuir* **22**, 3506-3513 (2006).
11. Savino, R., Paterna, D. & Favaloro, N. Buoyancy and Marangoni effects in an evaporating drop. *J. Thermophys. Heat Tr.* **16**, 562-574 (2002).
12. Yunker, P. J., Still, T., Lohr, M. A. & Yodh, A. G. Suppression of the coffee-ring effect by shape-dependent capillary interactions. *Nature* **476**, 308-311 (2011).
13. *Photonic Crystal*, <https://en.wikipedia.org/wiki/Photonic_crystal> (2015).
14. Joannopoulos, J. D., Villeneuve, P. R. & Fan, S. H. Photonic crystals: Putting a new twist on light. *Nature* **386**, 143-149 (1997).
15. *Colloidal_crystal*, <https://en.wikipedia.org/wiki/Colloidal_crystal> (2015).
16. Ge, J. P., He, L., Goebl, J. & Yin, Y. D. Assembly of Magnetically Tunable Photonic Crystals in Nonpolar Solvents. *J. Am. Chem. Soc.* **131**, 3484-+ (2009).
17. Arsenault, A. C. *et al.* From colour fingerprinting to the control of photoluminescence in elastic photonic crystals. *Nature Mater.* **5**, 179-184 (2006).
18. Busch, K. & John, S. Photonic band gap formation in certain self-organizing systems. *Phys. Rev. E* **58**, 3896-3908 (1998).
19. Comoretto, D. *et al.* Optical studies of artificial opals as 3D photonic crystals. *Mater Res Soc Symp P* **708**, 317-322 (2002).
20. Richel, A., Johnson, N. P. & McComb, D. W. Observation of Bragg reflection in photonic crystals synthesized from air spheres in a titania matrix. *Appl. Phys. Lett.* **76**, 1816-1818 (2000).
21. Huang, Y. *et al.* Colloidal photonic crystals with narrow stopbands assembled from low-adhesive superhydrophobic substrates. *J. Am. Chem. Soc.* **134**, 17053-17058 (2012).
22. Okuzono, T., Ozawa, K. & Doi, M. Simple model of skin formation caused by solvent evaporation in polymer solutions. *Phys. Rev. Lett.* **97**, 136103 (2006).
23. Atkins, P. & De Paula, J. *Atkins' Physical Chemistry*. 8 edn, Vol. 1 780 (Oxford University Press, Oxford, 2006).
24. De Gennes, P. G. Solvent evaporation of spin cast films: "crust" effects. *Eur. Phys. J. E* **7**, 31-34 (2002).
25. Brutin, D., Sobac, B., Loquet, B. & Sampol, J. Pattern formation in drying drops of blood. *J Fluid Mech* **667**, 85-95 (2011).
26. Sobac, B. & Brutin, D. Structural and evaporative evolutions in desiccating sessile drops of blood. *Phys. Rev. E* **84**, 011603 (2011).

27. *Viscosity*, <https://en.wikipedia.org/wiki/Viscosity> (2015).

28. Deegan, R. D. *et al.* Capillary flow as the cause of ring stains from dried liquid drops. *Nature* **389**, 827-829 (1997).

29. Morales, V. L. *et al.* Surfactant-mediated control of colloid pattern assembly and attachment strength in evaporating droplets. *Langmuir* **29**, 1831-1840 (2013).

30. Still, T., Yunker, P. J. & Yodh, A. G. Surfactant-induced Marangoni eddies alter the coffee-rings of evaporating colloidal drops. *Langmuir* **28**, 4984-4988 (2012).

31. Sempels, W., De Dier, R., Mizuno, H., Hofkens, J. & Vermant, J. Auto-production of biosurfactants reverses the coffee ring effect in a bacterial system. *Nature Commun.* **4**, 1757 (2013).

32. Pieranski, P. Two-Dimensional Interfacial Colloidal Crystals. *Phys. Rev. Lett.* **45**, 569-572 (1980).

33. Whetten, R. L. Two-dimensional crystallization: Express nanoparticle ordering. *Nature Mater.* **5**, 259-260 (2006).

34. Vogel, N., Goerres, S., Landfester, K. & Weiss, C. K. A Convenient Method to Produce Close- and Non-close-Packed Monolayers using Direct Assembly at the Air-Water Interface and Subsequent Plasma-Induced Size Reduction. *Macromol. Chem. Phys.* **212**, 1719-1734 (2011).

35. Anyfantakis, M., Geng, Z., Morel, M., Rudiuk, S. & Baigl, D. Modulation of the coffee-ring effect in particle/surfactant mixtures: the importance of particle-interface interactions. *Langmuir* **31**, 4113-4120 (2015).

36. Deng, Y., Zhu, X. Y., Kienlen, T. & Guo, A. Transport at the air/water interface is the reason for rings in protein microarrays. *J. Am. Chem. Soc.* **128**, 2768-2769 (2006).

37. Liu, Y., Goebl, J. & Yin, Y. Templated synthesis of nanostructured materials. *Chem. Soc. Rev.* **42**, 2610-2653 (2013).

38. Lang, L., Wu, D. & Xu, Z. Controllable fabrication of TiO(2) 1D-nano/micro structures: solid, hollow, and tube-in-tube fibers by electrospinning and the photocatalytic performance. *Chemistry* **18**, 10661-10668 (2012).

39. Nguyen, V. X. & Stebe, K. J. Patterning of small particles by a surfactant-enhanced Marangoni-Benard instability. *Phys. Rev. Lett.* **88** (2002).

40. Kajiya, T., Nishitani, E., Yamaue, T. & Doi, M. Piling-to-buckling transition in the drying process of polymer solution drop on substrate having a large contact angle. *Phys. Rev. E* **73** (2006).

41. Bigioni, T. P. *et al.* Kinetically driven self assembly of highly ordered nanoparticle monolayers. *Nature Mater.* **5**, 265-270 (2006).
